# Isolation and characterization of novel bat paramyxovirus B16-40 potentially belonging to the proposed genus *Shaanvirus*

**DOI:** 10.1038/s41598-018-30319-7

**Published:** 2018-08-22

**Authors:** Ji Yeong Noh, Dae Gwin Jeong, Sun-Woo Yoon, Ji Hyung Kim, Yong Gun Choi, Shien-Young Kang, Hye Kwon Kim

**Affiliations:** 1 Infectious Disease Research Center, Korea Research Institute of Bioscience and Biotechnology, Daejeon, Republic of Korea; 2 College of Veterinary Medicine, Chungbuk National University, Cheongju, 28644 Republic of Korea; 3 Bio-Analytical Science Division, University of Science and Technology (UST), Daejeon, Republic of Korea; 4 The Korean Institute of Biospeleology, Daejeon, Republic of Korea

## Abstract

The bat paramyxovirus B16-40 was first isolated in Korea in this study. Using the isolated virus, we could obtain not only genomic information, but also several biological characteristics of the virus. In the phylogenetic analysis, the virus was found to belong to the recently proposed genus *Shaanvirus*. Through sequence analyses and *in vitro* testing, the isolated virus was also found to have haemagglutinin-neuraminidase (HN) protein as one of the structural proteins. When mouse antiserum was generated against the isolated virus and tested, it was cross-reactive to human parainfluenza virus 1 in an indirect immunofluorescence assay but could not cross-neutralize human parainfluenza virus 1. In addition, the bat paramyxovirus B16-40 was not infectious in the mouse model. Collectively, this study provided basic information on further classification of the bat paramyxovirus B16-40 and related viruses in the proposed genus *Shaanvirus*.

## Introduction

Bats are considered a reservoir of severe emerging infectious diseases. Severe acute respiratory syndrome coronavirus (SARS-CoV), Middle East respiratory syndrome coronavirus (MERS-CoV), Nipah virus, Hendra virus, and Ebola virus are all thought to be bat-borne viruses^1,2^.

Notably, bats also host major mammalian paramyxoviruses from the family *Paramyxoviridae*, order *Monone-gavirales*^3,4^. While Henipaviruses (Nipah and Hendra viruses) in South East Asia and Australia are associated with fruit bats^5^, other paramyxoviruses have been detected not only in fruit bats but in insectivorous bats worldwide^6–9^. A potential pathway for Nipah virus transmission from bats to humans was found to be associated with a human-bat interface, specifically date palm sap shared by bats and humans^10^. In addition, serological evidence of possible human infection with a bat-originated paramyxovirus, Tioman virus^11^, reinforces the epidemiological role of bats in the emergence of pathogens such as paramyxoviruses in humans.

In addition to these bat paramyxoviruses with zoonotic potential, other new paramyxoviruses have been reported. These include several new mammalian paramyxoviruses such as Beilong virus and J virus, which remain unassigned under the family *Paramyxoviridae*^12^. Recent bat-associated paramyxoviruses were proposed to be grouped in a separate phylogenetic clade within a potentially separate genus such as *Shaanvirus*^13^ which was distantly related to *Jeilongvirus*^14^. In addition, novel strains of bat paramyxoviruses in diverse genera have been reported continuously^15–17^. Based on the recent papers, bat paramyxoviruses found worldwide to date have belonged to the genera *Rubulavirus*, *Morbillivirus*, *Henipavirus* and the unclassified proposed genera *Shaanvirus*. Expanded classifications for grouping newly identified viruses in bats can be accomplished by further studying the biological characteristics of novel paramyxoviruses as well as genome characterization^18^.

In this study, active surveillance was performed to reveal paramyxoviruses circulating in Korean bats. A total of 232 bat samples were collected at 48 sites in natural bat habitats and tested for the possible existence of paramyxoviruses.

## Results

### Identification of novel bat paramyxovirus B16-40

In this study, 232 bat samples were collected at 48 sites from natural bat habitats and tested for the possible existence of paramyxoviruses (Supplementary Table S1). Based on the RT-semi-nested PCR using the consensus paramyxovirus primers targeting the RdRp region^19^, five samples were positive and confirmed to be bat paramyxoviruses by sequencing. B16-6 was from a urine sample of *Miniopterus schreibersii* at BT cave in Hapcheon in March 2016, and B16-40 was from a feces sample of *Miniopterus schreibersii* at G cave in Danyang in April 2016. B16-148 and B16-154 were from feces samples of *Murina ussuriensis* at OJ cave in Youngwol and at S cave in Pyungchang in July 2016, respectively. B16-230 was from a urine sample of *Miniopterus schreibersii* at BLR cave in Seogwipo in December 2016. Among these, one paramyxovirus (B16-40) was successfully isolated.

**Figure 1:**
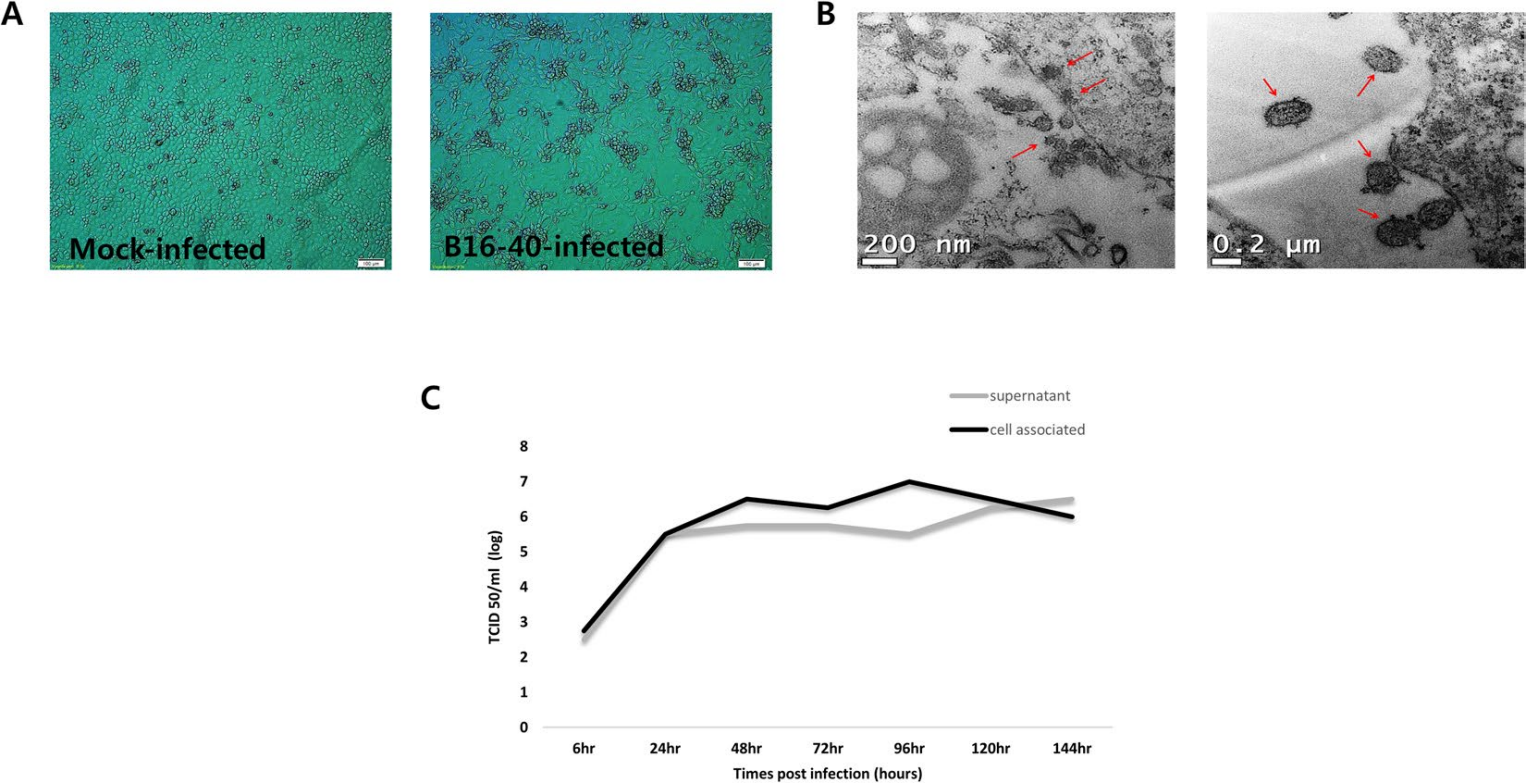
Virus culture and identification. (**A**) Mock-infected MARC 145 cells and bat paramyxovirus B16-40-infected MARC 145 cells showed cytopathic changes with light microscopy. (**B**) Bat paramyxovirus B16-40-infected MARC 145 cells imaged using transmission electron microscopy (TEM). (**C**) Viral growth kinetics for 6 days measured by infectious virus levels (TCID_50_/ml) in the supernatant and cell-associated sample. The time point of harvest was indicated as hours post infection.

The isolated virus, bat paramyxovirus B16-40, was isolated from the Korean bat *Miniopterus schreibersii*. The virus was successfully cultivated after three blind passages in the MARC-145 cell line (Fig. 1), which is a clone of the MA-104 cell line derived from kidney epithelial cells of an African green monkey^20^. Viral particles were observed in a pellet of infected MARC-145 cells under a transmission electron microscope (Fig. 1B). In terms of viral growth, infectious viruses were increased in the culture supernatant and infected cells from 24 hours post inoculation (hpi) and were consistently released for 144 hpi, as measured using tissue culture infectious dose 50 (TCID_50_) method (Fig. 1C).

### Infectivity in C57BL/6 mice

When 6-week-old female C57BL/6 mice were inoculated via the intranasal and intragastric routes with 10^5^ Tissue Culture Infectious Dose (TCID)_50_/mL or 10^2.5^ TCID_50_/mL of bat paramyxovirus B16-40, we found no evidence of infection, i.e. no viral shedding, histopathological findings, or seroconversion.

### Characterization of the bat paramyxovirus B16-40

Using high throughput sequencing (HiSeq. 2000 sequencing system based on the transcriptome *de novo* sequencing platform), 23,336 contigs totaling 8,010,635 base pairs (bps) with an average length of 343 bps were obtained. In the taxonomy annotation with MG-RAST, 12,522 contigs were annotated. While most of the annotated sequences associated with eukaryotes, 13 sequences were associated with family *Paramyxoviridae*. The 17,771 bases of viral genomic sequences were obtained by RT-PCR using primers designed based on the *Paramyxoviridae*-associated contigs and RACE sequencing.

**Figure 2:**
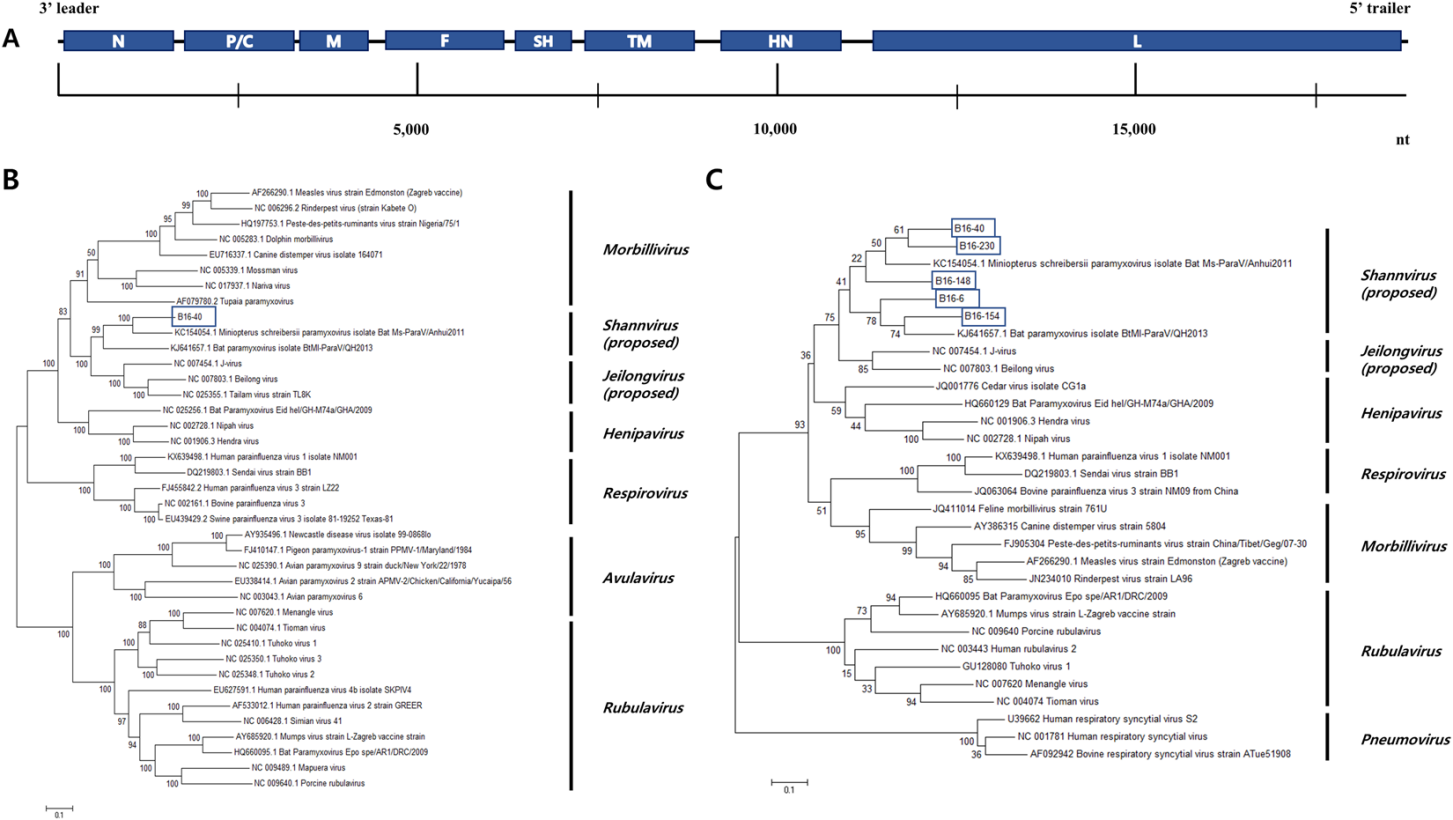
(**A**) Genome organization of the bat paramyxovirus B16-40 isolated in this study. The 3′ leader and 5′ trailer regions are lined, 8 ORF regions for the viral proteins are indicated by blue-filled boxes. N, nucleocapsid protein; P, phosphoprotein; M, matrix protein; F, fusion protein; SH, small hydrophobic protein; TM, transmembrane protein; HN, attachment haemagglutinin neuraminidase glycoprotein; L, large protein. (**B**) Phylogenetic analysis based on the genomic nucleotide sequence of the bat paramyxovirus B16-40. Reference viruses belonging to *Paramyxoviridae* were included and aligned by MAFFT. The phylogenetic tree was generated by the maximum-likelihood method with 1,000 replicates of bootstrap sampling and the Kimura 2-parameter model using MEGA 6. Bat paramyxovirus B16-40 isolated in this study is indicated with the blue box. (**C**) Phylogenetic analysis based on partial RNA-dependent RNA polymerase (RdRp) nucleotide sequences of bat paramyxoviruses detected in the Korean bats and other reference viruses belonging to *Paramyxoviridae*. The phylogenetic tree was generated by the maximum-likelihood method with 1,000 replicates of bootstrap sampling and the Kimura 2-parameter model using MEGA 6. Korean bat paramyxoviruses are indicated by blue boxes.

The genomic sequence of the virus encodes 8 Open Reading Frames (ORFs) (Fig. 2A), which is consistent with bat paramyxovirus strain Ms-ParaV/Anhui2011 (KC154054)^13^. In addition, maximum-likelihood trees showed that the bat paramyxovirus B16-40 strain was closely related to the bat paramyxovirus Ms-ParaV/Anhui2011 (KC154054) and BtMI-ParaV/QH2013 (KJ641657) strains rather than Beilong virus, J-virus, and Tailam virus (Figs 2B and S1). Even though the HN amino acids sequences were similar to those from viruses in the proposed genera *Shaanvirus*, it was also related to that of Sendai virus and human parainfluenza virus 1, which belong to a different genus, *Respirovirus* showing (Table 1).

**Table 1:** Nucleotide sequence (nt) and amino acid (aa) identities of bat paramyxovirus B16-40 with other viruses in *paramyxoviridae*.

Gene	*Ruvulavirus*		*Respiroviruses*				*Henipaviruses*				*Morbillivirus*		*Jeilongviruses*				proposed *Shaanviruses*			
	Mumps virus (AY685920.1)		Sendai virus (DQ219803.1)		Human parainfluenza virus 1 (KX639498.1)		Nipah virus (NC_002728.1)		Hendra virus (NC_001906.3)		Measles virus (AF266290.1)		J virus (NC_007454.1)		Beilong virus (NC_007803.1)		Bat paramyxovirus Bat MsNAParaV/Anhui2011 (KC154054)		Bat paramyxovirus BtMlNAParaV /QH2013 (KJ641657)	
	nt%	aa%	nt%	aa%	nt%	aa%	nt%	aa%	nt%	aa%	nt%	aa%	nt%	aa%	nt%	aa%	nt%	aa%	nt%	aa%
N	38	23.7	35.5	21.3	36.8	21.5	47	37.3	47.2	36.9	43.4	33.8	53.6	42	48.5	40.7	NA^a^	NA	NA	NA
P/V/W/C^b^	22.6(P)	10.2(P)	24.7(P)	9.2(P)	26.7(P)	9.5(P)	28.4(W)	14.8(P)	26.7(V)	14.2(P)	31.9(P)	18.6(P)	43.7(P)	24.5(P)	42.7(P)	27.5(P)	60.3(P)	45.3(P)	45.2(P)	24.4(P)
M	35	19.4	46.8	36.8	46.7	36.2	52.4	48.3	52.6	48.6	50.5	44.6	59.8	62.6	61.6	62.9	74.4	86.2	60.1	60.5
F	39.4	23.6	42.1	28.3	42.9	28.1	46.6	33.2	46.2	32.7	43	32.2	52.9	49.8	52.3	47.1	67.7	67.6	56.7	49.4
SH	10.9	2.4	NA	NA	NA	NA	NA	NA	NA	NA	NA	NA	10.3	4.4	12.8	7.7	58.4	50.2	24.2	12.7
TM	NA	NA	NA	NA	NA	NA	NA	NA	NA	NA	NA	NA	15.8	7.5	16	6.5	51.4	35.9	NA	NA
G (HNorH)^c^	36.5	22.3	39	28.8	40.9	27.8	35.6	17.6	35	17.6	29.7	13	37.5	27.9	36	27.8	58.6	56.8	40.2	27.1
L	40.4	27.2	46.1	38	48.4	38.5	52.8	45.2	52.9	45.5	52.5	46.4	60.1	57.6	59.1	57.8	59	65	54.6	54.3

^a^The data is not available. ^b^The P gene was expressed on polypeptide species (P/V/W/C). ^c^Glycoprotein; *Henipavirus*, *Jeilongvirus*, Hemagglutinin Neuraminidase protein; *Ruvulavirus*, *Respirovirus*, Hemagglutinin protein; *Morbillivirus*.

Based on the partial RNA-dependent RNA polymerase (RdRp) gene, the bat paramyxovirus isolates and other Korean bat paramyxoviruses belong to the genus *Shaanvirus*, with 68.8–79.0% nucleotide similarities (Fig. 2C). This suggests a single, closely related group of paramyxoviruses circulating in Korea.

**Figure 3:**
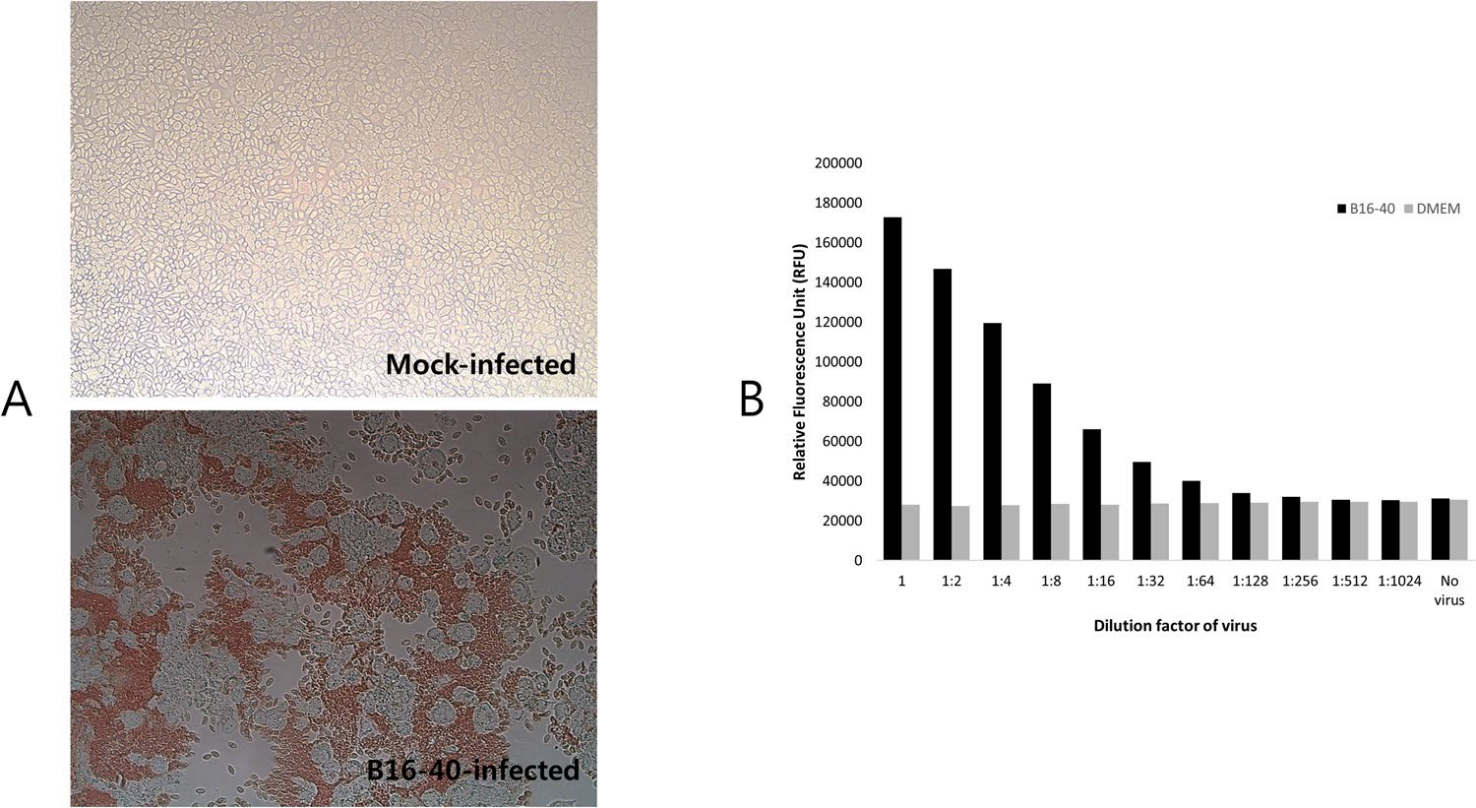
Haemagglutinating and neuraminidase activity of bat paramyxovirus B16-40. (**A**) Haemadsorption activity was determined with mock-infected and bat paramyxovirus B16-40-infected MARC-145 cells with 0.5% chicken RBCs. The bat paramyxovirus B16-40-infected cells showing cytopathic effects were surrounded by chicken RBCs. (**B**) Neuraminidase activity of the bat paramyxovirus B16-40 isolated in this study was determined by using the NA-Fluor assay. NA activity, which corresponds to the production of product (4-MU), was determined by comparing the average relative fluorescence units (RFU) from the non-virus control and the virus sample.

Consistent with the genetic findings, the bat paramyxovirus B16-40 was found to have haemagglutinin and neuraminidase activities in the haemadsorption assay (0.5% chicken red blood cells in PBS (pH 7.4)) and neuraminidase assay, respectively (Fig. 3).

### Cross-reaction and cross-neutralization tests

In the preliminary studies of mouse antisera (BALB/cA mice) against human respiratory syncytial virus A (KBPV-VR-41), human mumps virus (KBPV-VR-51) and human parainfluenza virus 1 (KBPV-VR-44), the antisera against human parainfluenza virus 1 (KBPV-VR-44) was only cross-reactive with the bat paramyxovirus B16-40 as determined using the immunofluorescence assay. Therefore, further cross-reactivity and cross-neutralizing tests between human parainfluenza virus 1 (KBPV-VR-44) and the bat paramyxovirus B16-40 were performed.

**Figure 4:**
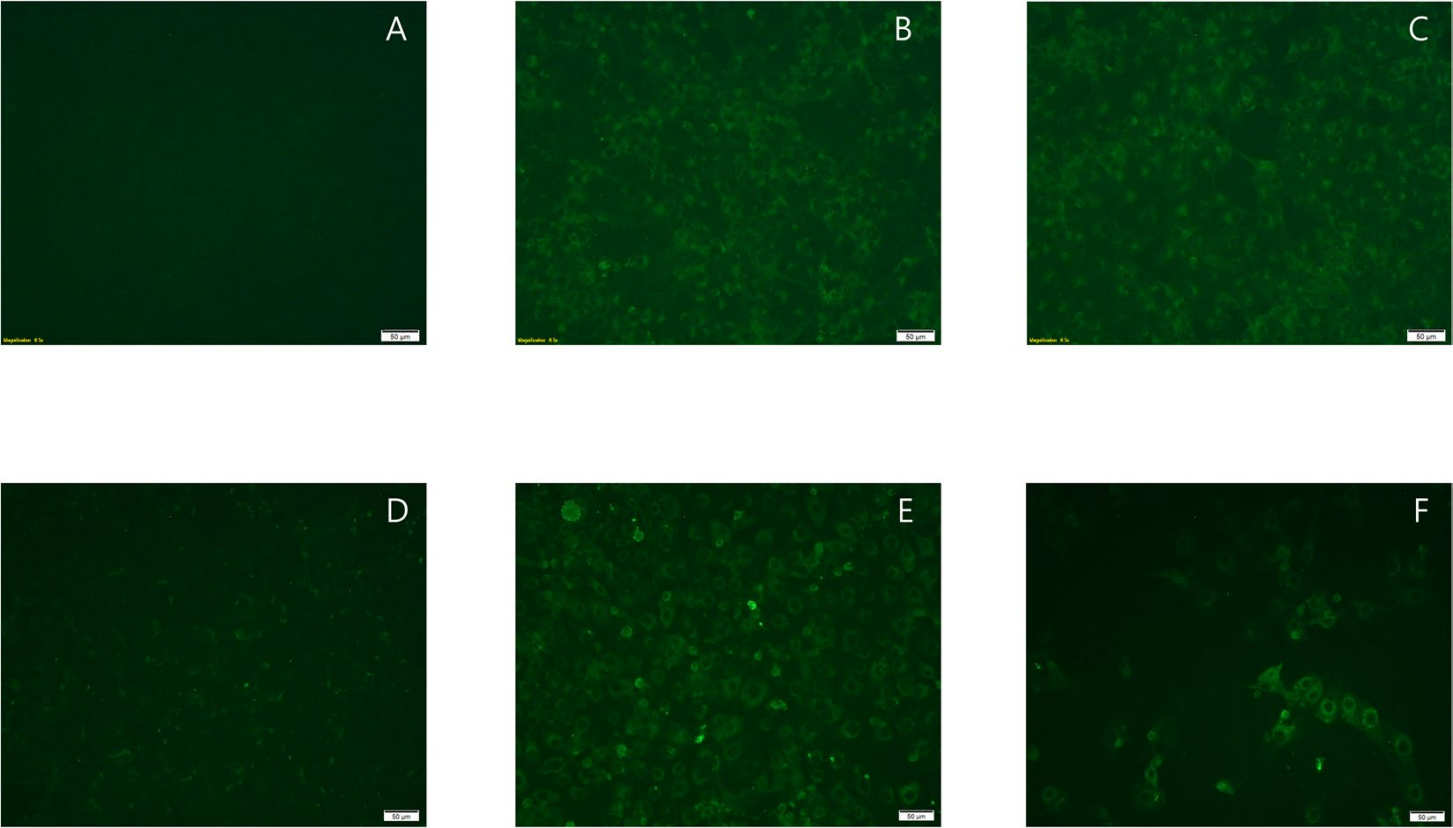
Cross-reactivity between bat paramyxovirus B16-40 and human parainfluenza virus 1 (KBPV-VR-44) was determined by fluorescent signal in an indirect immunofluorescence assay. Mock-infected (**A**) and bat paramyxovirus B16-40-infected (**B**,**C**) MARC-145 cells reacted with negative mouse serum diluted 40x (**A**) mouse antiserum against bat paramyxovirus B16-40 diluted 40x (**B**) and mouse antiserum against human parainfluenza virus 1 (KBPV-VR-44) diluted 40x. (**C**) Mock-infected (**D**) and human parainfluenza virus 1 (KBPV-VR-44)-infected (**E**,**F**) LLC-MK2 cells reacted with negative mouse serum diluted 40x (**D**) mouse antiserum against human parainfluenza virus 1 diluted 80x (KBPV-VR-44) (**E**) and mouse antiserum (pooled) against bat paramyxovirus B16-40 diluted 40x (**F**). All the images were equally adjusted to a brightness of +40 and a contrast of −40.

As shown in Table 2, partial cross-reactivities were observed between the two viruses in the indirect immunofluorescence assay, even though they belong to different genera. Among the six mouse sera against the bat paramyxovirus B16-40, two sera were cross-reactive to human parainfluenza virus 1 (KBPV-VR-44) with 10 and 40 as the end-point titers for the fluorescent signals. In addition, among three pooled sera (two mouse sera each) against human parainfluenza virus 1 (KBPV-VR-44), one pooled serum was cross-reactive to the bat paramyxovirus B16-40 with 40 as the end-point titer for the fluorescent signal (Table 2, Fig. 4).

**Table 2:** Cross-reaction and cross neutralization test between bat paramyxovirus (B16-40) and human parainfluenza virus 1 (KBPV-VR-44).

	Viral antigen	Individual Mouse ID	Viruses for Indirect immunofluorescence assay^a^		Viruses for Serum neutralization assay^b^	
			Bat Paramyxovirus (B16-40)	Human Parainfluenza virus 1	Bat Paramyxovirus (B16-40)	Human Parainfluenza virus 1
Antiserum	Bat Paramyxovirus (B16-40)	1	80	ND^d^	160	<10
		2	40	40	160	<10
		3	40	<10	160	<10
		4	80	ND	ND	ND
		5	<10	ND	20	ND
		6	10	10	80	<10
	Human Parainfluenza virus 1 (KBPV-VR-44)	1	40^c^	80	<10	40
		2		20	<10	40
		3	<10	20	<10	40
		4		ND	<10	40
		5	<10	20	<10	160
		6		ND	<10	20

^a^End-point titer was determined to the fluorescent signal in indirect immunofluorescence assay. ^b^End-point titer was determined to the inhibition of cytopathic effects in serum neutralization assay. ^c^The individual mouse sera were pooled. ^d^The data is not determined.

While the mouse antisera neutralized the homologous virus with 20–160 serum neutralizing titer, they did not neutralize the heterologous virus (Table 2).

## Discussion

The bat paramyxovirus B16-40 in this study was similar to viruses in the recently proposed genera *Shaanvirus* and *Jeilongvirus*. The genus *Jeilongvirus* includes Beilong virus, which was first isolated from human kidney mesangial cells^21^, and J virus, first isolated from mice^22^. These two viruses were only recently proposed to form *Jeilongvirus* as a distinct genus^14^. In addition, the newly discovered bat paramyxoviruses, Ms-ParaV/Anhui2011 and BtMl-ParaV/QH2013, were recently proposed to form the additional genus *Shaanvirus* due to their genomic length and sequence identity differences^13^. However, their taxonomy has not yet been confirmed by the International Committee on Taxonomy of Viruses (ICTV, https://talk.ictvonline.org). The proposed genera *Shaanvirus* and *Jeilongvirus* have two novel genes putatively encoding SH (small hydrophobic) and TM (transmembrane) proteins^12,13^. Two viral genes were also located between the fusion and haemagglutinin-neuraminidase genes of bat paramyxovirus B16-40. Further, their nucleotide and amino acids sequences were similar to the SH and TM genes identified from bat paramyxovirus strain Ms-ParaV/Anhui 2011. Therefore, the isolated bat paramyxovirus B16-40 might belong to the proposed genus *Shaanvirus* rather than *Jeilongvirus*.

The bat paramyxovirus B16-40 in this study was predicted to have a haemagglutinin-neuraminidase (HN) protein as an attachment glycoprotein in the sequence analysis. Consistent with the genetic findings, the bat paramyxovirus B16-40 was found to have haemagglutinin and neuraminidase activities in the haemadsorption assay (0.5% chicken red blood cells in PBS (pH 7.4)) and neuraminidase assay, respectively (Fig. 2A,B). Notably, even though the HN amino acids sequences were similar to those from viruses in the proposed genera *Shaanvirus*, it was also related to that of Sendai virus and human parainfluenza virus 1, which belong to a different genus, *Respirovirus* (Table 1). Maximum-likelihood tree analysis based on the full HN amino acid sequences also showed that the bat paramyxovirus B16-40 was related to viruses in the genus *Respirovirus* (Supplementary Fig. S1). Therefore, the B16-40 HN amino acid sequence was compared to that of human parainfluenza virus 1 (KBPV-VR-44), and six regions that have at least six conserved amino acids were found. These regions were located on the sialidase superfamily domain and were structurally gathered together (Supplementary Fig. S2). Among the seven neuraminidase active sites (R, D, E, R, R, Y, and E^23^), two of these (E at the third site and Y) were located within the conserved amino acid sequences. These bioinformatics findings might indicate that the HN proteins of bat paramyxovirus B16-40 and human parainfluenza virus 1 (KBPV-VR-44) are genetically related, even though they are in different genera. Further study on their antigenic and evolutionary relationship should be followed up.

Notably, in a previous study, bat serum (diluted 1/40) from African wild bat, *Micropteropus pusillus* was reactive with human parainfluenza virus 1 in an indirect immunofluorescence assay^3^. There may be two possibilities to explain this. First, the African bat might have been infected with a human parainfluenza virus 1-related virus in the genus *Respirovirus* that has not yet been found. However, as there has been no evidence of bat paramyxoviruses related to the genus *Respirovirus* in recent studies^13,15,16^, the first possibility seems low. Second, the bat might have been infected by other bat paramyxoviruses which were cross-reactive with human parainfluenza virus 1. In fact, in this study, when mouse antisera were made and tested against bat paramyxovirus B16-40 and human parainfluenza virus 1 (KBPV-VR-44), the two viruses were partially cross-reactive to each other in an indirect immunofluorescence assay. This result provided an evidence that there could be cross-reactivity between viruses belonging to different genera. Therefore, the bat paramyxovirus B16-40-related viruses circulating in bats might be one of the reasons why the wild bat sera was reactive with human parainfluenza virus 1 in the previous study.

However, we could not find the cross-neutralization between bat paramyxovirus B16-40 and human parainfluenza virus 1. Based on the recent studies, cross-neutralization was found between the viruses in the same genus of paramyxoviruses: Sendai virus and human parainfluenza virus 1, which both belong to the genus *Respirovirus*^24^, and human mumps virus and African bat mumps virus, which both belong to the genus *Rubulavirus*^25^. Further, the engaged proteins in the cross-reactivity between bat paramyxovirus B16-40 and human parainfluenza virus 1 had not been revealed. Therefore, although bat paramyxovirus B16-40 in this study was cross-reactive with human parainfluenza virus 1 in indirect immunofluorescence assay, it is difficult to say that the bat paramyxovirus B16-40 was antigenically related with human parainfluenza virus 1.

Since a number of paramyxoviruses have been newly found around world, their classification has been difficult due to the limited criteria, such as sequence alone^18^. Additional input from the biological context may be helpful. In this study, a bat paramyxovirus B16-40 belonging to the proposed genus *Shaanvirus* was first isolated. Through virus isolation, we could obtain not only nucleotide sequence information, but also several biological characteristics of the virus. Its putative HN protein was demonstrated to have haemagglutinin and neuraminidase activities. Mouse antisera against the bat paramyxovirus B16-40 was also cross-reactive to human parainfluenza virus 1 in the indirect immunofluorescence assay, but could not cross-neutralize human parainfluenza virus 1. Additionally, the bat paramyxovirus B16-40 was not infectious in 6-week-old female C57BL/6 mice. Therefore, this study provided basic information on further classification of the bat paramyxovirus B16-40 and related viruses in the proposed genus *Shaanvirus*.

## Methods

### Ethical approval

All animal experiments were conducted at the Korea Research Institute of Bioscience and Biotechnology (KRIBB, Daejeon, Korea), and were approved by and conducted in accordance with the guidelines of the Institutional Animal Care and Use Committee of KRIBB (Approval No. KRIBB-AEC-16181 and KRIBB-AEC-17064).

### Samples

In 2016, a total of 232 samples were collected at 48 sites in natural bat habitats. Most samples were collected as fresh guano under the colony of bats. In some cases, bats were captured by a fine mesh net to collect fresh feces as well as oropharyngeal and urine swabs, and the animals were then immediately released. The collected samples were placed into transport medium (Noble Bio. Co. Ltd) in a 10% suspension and were transported to the lab (BSL2) for further analysis. The major bat species at the collection sites were determined based on morphology and on previous data from bats’ roosting sites^26^. These species were *Rhinolophus ferrumequinum*, *Murina leucogaster*, *Miniopterus*, *Murina ussuriensis*, *Eptesicus serotinus*, *Myotis bombinus*, *Myotis macrodactylus*, *Hypsugo alaschanicus*, and *Myotis daubentoni* (Supplementary Table S1).

### RT-semi-nested PCR and virus isolation

RNA extracted from bat samples was tested by RT-semi-nested PCR using consensus paramyxovirus primers targeting the RdRp region^19^. The nucleotide sequences of the positive samples were determined by Cosmogenetech Co. Ltd. Five of the 232 bat samples were confirmed positive to the bat paramyxoviruses by sequencing. Virus isolation was performed from the RT-semi-nested PCR-positive samples including feces, urine, and oropharyngeal swabs from bats. Each bat sample was diluted 10-fold with fresh Dulbecco Modified Eagle Medium (DMEM) and filtered with a 0.2-μm filter before being inoculated into the MARC 145 cell line, a clone from the MA-104 cell line from kidney epithelial cells of an African green monkey^20^; blind passaging was performed up to three times. The cytopathic effect (CPE) was confirmed in cells infected with one sample (B16-40) of five PCR-positive samples. Next, a monolayer of MARC 145 was infected with bat paramyxovirus (B16-40) at a multiplicity of infection (MOI) of 0.1. Two days post-infection, the infected cells were harvested and pelleted. The infected cell pellet was fixed with 2.5% glutaraldehyde in PBS (pH 7.4) for 2 hours and sent for transmission electron microscopy (TEM) to the Advanced Analysis Center in the Korean Institute of Science and Technology in Korea. Briefly, after washing with PBS the cells were post-fixed in 2% osmium tetroxide and dehydrated in an ethanol series (70%, 80%, 85%, 90%, 95%, and 100% (two times)). Then, the sample was transited with 100% propylene oxide and embedded in Epoxy resin. The embedded sample was sectioned into 70–80 nm sections with an ultramicrotome (Ultra Cut C; Leica), stained with 2% uranyl acetate and lead citrate on a copper grid, and examined using TEM mode on a CryoTecnai F20 (FEI Ltd., USA).

### Virus growth and titration

To observe growth kinetics of the isolated bat paramyxovirus B16-40, 1 × 10^5^/mL of MARC-145 cells were seeded to 25 cm^2^ tissue culture flasks in DMEM (5% FBS) and incubated for 24 hours to create a monolayer. The virus was then inoculated to the monolayer of MARC-145 cells at a MOI of 0.1 and maintained in DMEM (2.5% FBS) for 6 days. Every 24 hours (6 hours on day 0), culture supernatant was collected for viral titration. The remaining cell layer was washed three times with PBS (pH 7.4), and 1 mL of fresh DMEM was added to release the cell-associated viruses and subsequent freezing and thawing. The collected culture supernatant and freeze-thawed cell lysate were centrifuged at 3,000 × g for 10 minutes to remove cell pellets and then used for viral titration.

The infectious viruses were quantified from the prepared samples using the tissue culture infectious dose 50(TCID_50_) method. Briefly, the prepared samples were 10-fold serially diluted and inoculated to monolayers of MARC-145 cells in 4 wells. The highest dilution showing 50% of viral infection was obtained by observing cytopathic effects with a light inverted microscope (IX71S1F-3, OLYMPUS CORPORATION). The TCID_50_ was calculated using the Spearman-Karber method.

### Genomic sequencing and analyses

A virus isolate showing cytopathic effects (CPEs) was filtered through a 0.2 μm filter and ultra-centrifuged at 250,000 × g for 1 hour. Each pellet was suspended in 500 µL of 1x digestion buffer (Turbo DNA Free Kit; Ambion) and treated with Turbo DNase; the suspension was incubated at 37 °C for 30 min. The RNA from the suspension was isolated using the Trizol LS reagent according to the manufacturer’s instructions (Ambion). The extracted RNA was submitted to Macrogen (Seoul, Korea) for high throughput sequencing in a HiSeq. 2000 sequencing system based on the transcriptome *de novo* sequencing platform. The obtained viral contigs were analysed and annotated by the MG-RAST server^27^. The cut-off for the annotation was 10^−5^ maximum e-value, 60% minimum percentage identity, and 15 for minimum alignment length. The viral genomic sequences were obtained by RT-PCR using primers based on the *Paramyxoviridae*-associated contigs obtained by high throughput sequencing. Additionally, 3′ and 5′ end sequences were obtained by RACE sequencing based on PCR using the adapter-oligo dTVN primer from Bionics Co. Ltd.

For human parainfluenza virus 1 (KBPV-VR-44), the 1,992 bases of the HN gene were obtained by RT-nested PCR following the previously published method^28^. The obtained genomic RNA sequences and RT-PCR-positive amplicons were further analysed with related sequences in GenBank using MAFFT^29^ and MEGA version 6^30^.

Genomic sequence data generated in this study have been deposited in GenBank under accession number MG230624. The full HN sequence of human parainfluenza virus 1 (KBPV-VR-44) has also been deposited in GenBank under accession number MG255129. The HN amino acid sequences of human parainfluenza virus 1 (KBPV-VR-44) and bat paramyxovirus were further compared with the BioEdit program^31^. Putative HN protein structures were predicted using the Phyre2 website^32^ and visualized with the UCSF Chimera 1.11.2 program^33^.

### Haemadsorption and neuraminidase assays

To investigate the activity of the HN protein, the haemadsorption assay was used. Monolayer cultures of MARC 145 cells in 96-well plates were inoculated with the bat paramyxovirus B16-40 at an MOI of 0.1. After 48 hours, the cells were gently washed with PBS and incubated for 1 hour with 0.5% chicken red blood cells (RBCs) in PBS (pH 7.4) at room temperature. Then, the cells were washed with PBS twice to remove unbound RBCs and observed under a microscope. In addition, the neuraminidase assay was performed with the viral isolate using the NA-Fluor Influenza Neuraminidase Assay Kit (Thermo) according to the manufacturer’s protocol. The virus was diluted 2-fold with the NA-Fluor 2× assay buffer in a black 96-well plate and incubated for 1 hour at 37 °C with the NA-Fluor substrate. After adding the stop solution, the plate was read at the excitation and emission wavelengths of 355 nm and 460 nm, respectively, using a PerkinElmer VICTOR2 fluorometer.

### Experimental inoculation of bat paramyxovirus B16-40 in mice

A total of 44 6-week-old female C57BL/6 mice maintained under specific-pathogen-free (SPF) conditions were divided into two groups of negative controls (n = 4 each) and four inoculation groups (n = 9 each). The inoculation groups were inoculated with 400 µL of 10^5^ TCID_50_ or 10^2.5^ TCID_50_ of the bat paramyxovirus B16-40 via intra-gastric administration routes, and 30 µl of 10^5^ TCID_50_ or 10^2.5^ TCID_50_ of the bat paramyxovirus B16-40 via the intranasal administration routes. Each inoculation was conducted twice with 1 week in between. Weight loss was monitored daily for up to 2 weeks. The lungs, liver, brain, and intestine were harvested at 7 or 14 days post-challenge, fixed with 4% paraformaldehyde in PBS, pH = 7.2, and submitted to BioLead Inc., Korea, for histopathological examination. Additionally, RNA was extracted from each tissue and from oral swabs and fecal samples collected daily for 7 days post-challenge. The extracted RNA was tested using RT-semi-nested PCR with the consensus paramyxovirus primers^19^. In addition, real time PCR was performed with newly designed primers and probes targeting regions of the membrane and fusion protein. Briefly, the designed primers were as follows:

forward primer: PVM-F5′-CCCAGGAGTATGGTTATCAAGTGAGG-3′; reverse primer: PVM-R 5′-TCCATTGGGCTCTCTTTGTTTGC-3′; Taqman probe: PVM-P 5′-FAM-CCCATCCCAGACCAGCCACCAGACCC-TAMRA-3′

Real time RT-PCR was performed as follows: Reverse transcription at 45 °C for 10 min, followed by PCR −95 °C for 5 min, cycling 40 times at 95 °C for 10 sec, and 60 °C for 30 sec. Using the 10-fold diluted virus (bat paramyxovirus B16-40), standard curves were performed for every reaction.

### Generation of mouse antisera against bat paramyxovirus B16-40 and human paramyxoviruses

We next observed preliminary cross-reactivity in indirect immunofluorescence assay between several human viruses belonging to the family *Paramyxoviridae* and the bat paramyxovirus B16-40. 6-week-old female BALB/cA mice maintained under SPF conditions were intramuscularly inoculated twice, with 2 weeks in between, with a 1:1 ratio mixture of AddaVax adjuvant and live virus stock (bat paramyxovirus B16-40, human respiratory syncytial virus A (KBPV-VR-41), human mumps virus (KBPV-VR-51), and human parainfluenza virus 1 (KBPV-VR-44), respectively, from the Korea Bank for Pathogenic Viruses. Two weeks after the last immunization, antisera against the virus were obtained by cardiac puncture for further studies related to cross-reactivity in indirect immunofluorescence assay. Mouse antisera against the bat paramyxovirus B16-40 (10^5.75^ TCID_50_/mL) and human parainfluenza virus 1 (KBPV-VR-44, 10^5.75^ TCID_50_/mL) were further compared through serum titration using the indirect immunofluorescence assay and serum neutralization test.

### Indirect immunofluorescence assay

To evaluate the level of serological cross-reactivity, monolayers of MARC 145 and LLC-MK2 cells in 96-well plates were inoculated with 200 TCID_50_ of the bat paramyxovirus B16-40 and human parainfluenza virus 1 (KBPV-VR-44), respectively, or were mock-infected. Mock-infected cells were used as a negative control for non-specific binding of the mouse serum. The plates were incubated at 37 °C for 48 hours. Then the supernatant was discarded and cells were washed with PBS (pH 7.4). The washed cells were fixed with 100% ethanol for 1 hour at −20 °C and rehydrated with PBS (pH 7.4) for 10 min at room temperature. Primary antibodies used in the experiments were the mouse antisera against bat paramyxovirus B16-40 and human parainfluenza virus 1 (KBPV-VR-44). The sera were diluted with 50% culture supernatants from the cell lines in fresh DMEM; the corresponding mock-infected cells were treated in the same way as the target virus culture. Then, the sera were serially diluted 2-fold from an initial dilution of 1:10 and added to the fixed infected or mock-infected cells and incubated for 1 hour at room temperature. The plates were washed three times with PBS containing 0.05% Tween 20 (PBS-T, pH 7.4). FITC-conjugated goat anti-mouse IgG secondary antibody (Abcam; cat No. ab6785) was diluted 1:2000 with the same serum diluent and was added to the plates and incubated for 1 hour at room temperature. After three washes with PBS-T, the plates were viewed with a fluorescence microscope (IX71S1F-3, OLYMPUS CORPORATION).

### Serum neutralization test

The mouse serum samples were inactivated at 56 °C for 30 min. They were then diluted 2-fold from an initial dilution of 1:10 in 96-well plates and mixed with 200 TCID_50_ of each virus. The plates were then incubated at 37 °C for 1 hour. The virus-serum mixtures for the bat paramyxovirus B16-40 and human parainfluenza virus 1 (KBPV-VR-44) were added to monolayer cultures of MARC 145 and LLC-MK2 cells, respectively, and incubated at 37 °C for 1 hour. The MARC 145 cells and LLC-MK2 cells were then washed once with PBS (pH 7.4) and maintained in DMEM (2.5% FBS) for 5 days. After incubation, the plates were examined for CPE.

### Data availability

The datasets generated during and/or analysed during the current study are available from the corresponding author on reasonable request.

## Electronic supplementary material

Supplement Information

## References

[1] Han H-J (2015). Bats as reservoirs of severe emerging infectious diseases. Virus research.

[2] Kim H (2016). Detection of Severe Acute Respiratory Syndrome‐Like, Middle East Respiratory Syndrome‐Like Bat Coronaviruses and Group H Rotavirus in Faeces of Korean Bats. Transboundary and emerging diseases.

[3] Drexler JF (2012). Bats host major mammalian paramyxoviruses. Nature communications.

[4] Amarasinghe GK (2017). Taxonomy of the order Mononegavirales: update 2017. Archives of virology.

[5] Aljofan M (2013). Hendra and Nipah infection: emerging paramyxoviruses. Virus research.

[6] Barr J (2015). Isolation of multiple novel paramyxoviruses from pteropid bat urine. Journal of General Virology.

[7] Kurth A (2012). Novel paramyxoviruses in free-ranging European bats. Plos one.

[8] Mortlock M (2015). Novel paramyxoviruses in bats from sub-Saharan Africa, 2007–2012. Emerging infectious diseases.

[9] Wilkinson DA (2012). Identification of novel paramyxoviruses in insectivorous bats of the Southwest Indian Ocean. Virus research.

[10] Islam MS (2016). Nipah virus transmission from bats to humans associated with drinking traditional liquor made from date palm sap, Bangladesh, 2011–2014. Emerging infectious diseases.

[11] Yaiw KC (2007). Serological evidence of possible human infection with Tioman virus, a newly described paramyxovirus of bat origin. Journal of Infectious Diseases.

[12] Jack PJ, Boyle DB, Eaton BT, Wang L-F (2005). The complete genome sequence of J virus reveals a unique genome structure in the family *Paramyxoviridae*. Journal of virology.

[13] Wu Z (2016). Deciphering the bat virome catalog to better understand the ecological diversity of bat viruses and the bat origin of emerging infectious diseases. The ISME journal.

[14] Audsley MD (2016). The immune evasion function of J and Beilong virus V proteins is distinct from that of other paramyxoviruses, consistent with their inclusion in the proposed genus Jeilongvirus. Journal of General Virology.

[15] Rizzo F (2017). Coronavirus and paramyxovirus in bats from Northwest Italy. BMC veterinary research.

[16] Bourgarel, M. *et al*. Circulation of Alphacoronavirus, Betacoronavirus and Paramyxovirus in Hipposideros bat species in Zimbabwe. *Infection*, *Genetics and Evolution* (2018). 10.1016/j.meegid.2018.01.007 PMC7106086 29331670

[17] Pauly M (2017). Novel Alphacoronaviruses and paramyxoviruses Cocirculate with type 1 and severe acute respiratory system (SARS)-related Betacoronaviruses in Synanthropic bats of Luxembourg. Applied and Environmental Microbiology.

[18] Rima, B. *et al*. Problems of classification in the family *Paramyxoviridae*. *Archives of virology*, 1–10 (2018). 10.1007/s00705-018-3720-2 PMC6309968 29372404

[19] Tong S, Chern S-WW, Li Y, Pallansch MA, Anderson LJ (2008). Sensitive and broadly reactive reverse transcription-PCR assays to detect novel paramyxoviruses. Journal of clinical microbiology.

[20] Kim H, Kwang J, Yoon I, Joo H, Frey M (1993). Enhanced replication of porcine reproductive and respiratory syndrome (PRRS) virus in a homogeneous subpopulation of MA-104 cell line. Archives of virology.

[21] Li Z (2006). Beilong virus, a novel paramyxovirus with the largest genome of non-segmented negative-stranded RNA viruses. Virology.

[22] Jun M, Karabatsos N, Johnson R (1977). A new mouse paramyxovirus (J virus). Aust. J. Exp. Biol. Med. Sci.

[23] Yuan P (2005). Structural studies of the parainfluenza virus 5 hemagglutinin-neuraminidase tetramer in complex with its receptor, sialyllactose. Structure.

[24] Adderson E (2015). Safety and immunogenicity of an intranasal Sendai virus-based human parainfluenza virus type 1 vaccine in 3-to 6-year-old children. Clinical and Vaccine Immunology.

[25] Katoh H (2016). Cross-neutralization between human and African bat mumps viruses. Emerging infectious diseases.

[26] Han, S., Jung, C. W., Choi, Y. G. & Kim, S. S. Sounds of the Bats in Korea. *National Institute of Biological Resources Press* (2012).

[27] Meyer F (2008). The metagenomics RAST server–a public resource for the automatic phylogenetic and functional analysis of metagenomes. BMC bioinformatics.

[28] Košutić-Gulija T, Slovic A, Ljubin-Sternak S, Mlinarić-Galinović G, Forčić D (2016). A study of genetic variability of human parainfluenza virus type 1 in Croatia, 2011–2014. Journal of medical microbiology.

[29] Katoh K, Standley DM (2013). MAFFT multiple sequence alignment software version 7: improvements in performance and usability. Molecular biology and evolution.

[30] Tamura K, Stecher G, Peterson D, Filipski A, Kumar S (2013). MEGA6: molecular evolutionary genetics analysis version 6.0. Molecular biology and evolution.

[31] Hall, T. A. In *Nucleic acids symposium series*. 95–98 ([London]: Information Retrieval Ltd., c1979–c2000) (1999).

[32] Kelley LA, Mezulis S, Yates CM, Wass MN, Sternberg MJ (2015). The Phyre2 web portal for protein modeling, prediction and analysis. Nature protocols.

[33] Pettersen EF (2004). UCSF Chimera—a visualization system for exploratory research and analysis. Journal of computational chemistry.

