# Supplementary material for: Isolation and characterization of novel bat paramyxovirus B16-40 potentially belonging to the proposed genus Shaanvirus
Source: Sci Rep. 2018 Aug 22;8:12533. doi: 10.1038/s41598-018-30319-7 (PMC6105681; doi:10.1038/s41598-018-30319-7)
Supplement: Supplementary file 1 — Supplement Information [file 41598_2018_30319_MOESM1_ESM.pdf]

Isolation and characterization of novel bat paramyxovirus B16-40 potentially belonging to the proposed genus *Shaanvirus*

Ji Yeong Noh[1,2,*], Dae Gwin Jeong[1,3, *], Sun-Woo Yoon[1,3], Ji Hyung Kim[1], Yong Gun Choi[4], Shien-Young Kang[2,**], Hye Kwon Kim[1,**]

[1]*Infectious Diseases Research Center, Korea Research Institute of Bioscience and Biotechnology, Daejeon, Republic of Korea*

[2]*College of Veterinary Medicine, Chungbuk National University, Cheongju 28644, Republic of Korea*

[3]*Bio-Analytical Science Division, University of Science and Technology (UST), Daejeon, Republic of Korea*

[4]*The Korean Institute of Biospeleology, Daejeon, Republic of Korea*

[*] These authors contributed equally to this work

[**] These authors are co-corresponding authors

Address correspondence to Shien-Young Kang, skykang@cbu.ac.kr; Hye Kwon Kim, khk1329@kribb.re.kr

Supplementary table S1. Bat samples information

Cases	Region	Site name	Collected date	Major bat species	No. of samples		
					feces	urine	oral swab
1	Munkyung	HG abandoned mine	16-Mar-16	Miniopterus	3		
2	Hapchoen	BT cave	16-Mar-16	Miniopterus	2	1	
			18-May-16		2	2	8
			28-Aug-16		3	1	2
			08-Oct-16		2	1	
3	Jeju	USJ cave	21-Mar-16	Rhinolophus ferrumequinum	1	1	2
4	Jeju	MJ cave	21-Mar-16	Rhinolophus ferrumequinum	1		3
5	Seogwipo	artificial cave	22-Mar-16	Miniopterus	1	1	
			18-Aug-16		2		1
6	Jeju	artificial cave	23-Mar-16	Rhinolophus ferrumequinum, Myotis bombinus	1		1
7	Seogwipo	HDR cave	23-Mar-16	Miniopterus	1	1	1
			14-Dec-16	Rhinolophus ferrumequinum	1		3
8	Jungsun	HPDPN cave	20-Mar-16	Rhinolophus ferrumequinum	1		
9	Jindo	SJ cave	25-Mar-16	Rhinolophus ferrumequinum, Myotis macrodactylus	2	2	2
10	Moan	UJD cave	26-Mar-16	Rhinolophus ferrumequinum		1	1
11	Moan	SP cave	26-Mar-16	Rhinolophus ferrumequinum, Myotis macrodactylus		2	2
12	Moan	IS cave	26-Mar-16	Rhinolophus ferrumequinum		1	1
13	Danyang	G cave	10-Apr-16	Miniopterus, Murina ussuriensis	2		
			16-Apr-16		2		
			27-Jun-16		11	1	4
			13-Oct-16		3		1
14	Danyang	G cave	17-Apr-16	Rhinolophus ferrumequinum, Murina leucogaster		1	2
15	Jeju	SC cave	28-Apr-16	Rhinolophus ferrumequinum		1	1
16	Jeju	MSB cave	28-Apr-16	Rhinolophus ferrumequinum	1		
			19-Aug-16				2

			05-Nov-16			1	1
17	Youngju	deserted house	10-May-16	unknown	1		
18	Munkyung	BGA cave	18-May-16	Rhinolophus ferrumequinum	1		3
19	Munkyung	BGS cave	18-May-16	Miniopterus	4	1	3
20	Hampeong	JC cave	25-May-16	Rhinolophus ferrumequinum	2	2	1
21	Munkyung	UL cave	29-May-16	Rhinolophus ferrumequinum, Murina leucogaster	2	2	2
22	Jeju	GR cave	09-Jun-16	Miniopterus	1	1	2
23	Kyungju	SC bridge	15-Jun-16	Hypsugo alaschanicus	3		
24	Jungsun	SM cave	19-Jun-16	Murina ussuriensis	3		1
25	Danyang	HSA bridge	27-Jun-16<br>18-Jul-16<br>11-Sep-16<br>02-Oct-16	Eptesicus serotinus	4<br>2<br>2<br>1		
26	Chungju	CS cave	28-Jun-16	Rhinolophus ferrumequinum	5		
27	Danyang	HA bridge	28-Jun-16	Eptesicus serotinus	3		2
28	Danyang	SM cave	28-Jun-16	Rhinolophus ferrumequinum	1		
29	Donghae	SH temple	13-Jul-16	Hypsugo alaschanicus	2		
30	Yeongwol	GS cave	16-Jul-16<br>24-Sep-16	Murina ussuriensis	2<br>2		
31	Pyungchang	BR cave	16-Jul-16	Murina ussuriensis	2		
32	Yeongwol	WS bridge	19-Jul-16	Eptesicus serotinus	5		1
33	Yeongwol	OJ cave	19-Jul-16	Rhinolophus ferrumequinum, Murina ussuriensis	12	1	2
34	Pyungchang	S cave	20-Jul-16	Rhinolophus ferrumequinum, Murina ussuriensis	5		
35	Pyungchang	SR cave	20-Jul-16	unknown	1		
36	Jeju	HH cave	19-Aug-16	Rhinolophus ferrumequinum	1		1
37	Jeju	KNBLM cave	20-Aug-16<br>04-Nov-16	Rhinolophus ferrumequinum, Myotis macrodactylus	2	2	3
38	Jungsun	WP cave	24-Sep-16	Rhinolophus ferrumequinum	1	1	1
39	Jungsun	HM cave	24-Sep-16	Rhinolophus ferrumequinum		1	1
40	Jungsun	BR cave	25-Sep-16	Rhinolophus ferrumequinum	1	1	1

41	Jungsun	GAU cave	16-Oct-16	Rhinolophus ferrumequinum	1		1
42	Jungsun	GJ cave	22-Oct-16	Rhinolophus ferrumequinum		1	1
43	Jungsun	YM cave	23-Oct-16	Rhinolophus ferrumequinum, Myotis daubentoni	1	1	1
			20-Nov-16		3	1	1
44	Jeju	WH cave	05-Nov-16	Miniopterus	3		
45	Pyungchang	GCS cave	07-Dec-16	Rhinolophus ferrumequinum		1	2
46	Okchun	GJR cave	11-Dec-16	Rhinolophus ferrumequinum		1	1
47	Seogwipo	BLR cave	05-Nov-16	Rhinolophus ferrumequinum, Miniopterus	1		
			14-Dec-16			2	2
48	Sangju	SJY cave	25-Dec-16	Rhinolophus ferrumequinum		1	1
			total			232	

The abbreviations list: HG: Ho Gye, BT: Be Ti, USJ: Us San Jeon, MJ: Man Jang, HDR: Han Deu Reu, HPDPN: Hwa Pyo Dong Pi Nan, SJ: Sib Ja, UJD: U Jeog Dong, SP: Seog Po, IS: I Seong, G: Geum, G: Gom, SC: So Cheon, MSB: Myo San Bong, BGA: Bu Gog Am, BGS: Bu Gog Sus, JC: Jeong Chang, UL: U Lo, GR: Gu Rin, SC: Sin Cheon, SM: Saem Mul, HSA: Ha Seon Am, CS: Cheong Seog, HA: Heung Am, SM: Seol Ma, SH: Sam Hwa, GS: Go Ssi, BR: Baeg Ryong WS: Wa Seog, OJ: Ong Jeong, S: Ssang, SR: Sa Rang, HH: Hwa Heul, KNBLM: Gim Nyeong Bil Le Mos, WP: Wol Pyeong, HM: Haeng Mae, BR: Bi Ryong, GAU: Gul A U, GJ: Gol Ji, YM: Ya Mi, WH: Wa Heul, GCS: Gwang Cheon Seon, GJR: Gwang Ju Ri, BLR: Beol La Ri, SJY: Sang Ju Yong

Supplementary figure S1. Phylogenetic analysis based on the amino acid sequences of the M (A), F (B), and HN (C) proteins of the bat paramyxovirus B16-40 and other reference viruses belonging to Paramyxoviridae. The phylogenetic tree was generated by the maximum-likelihood method with 1,000 replicates of bootstrap sampling and the Jones-Taylor-Thornton (JTT) model using MEGA.[1,2] The bat paramyxovirus B16-40 isolated in this study is denoted by the red box.

A. Matrix

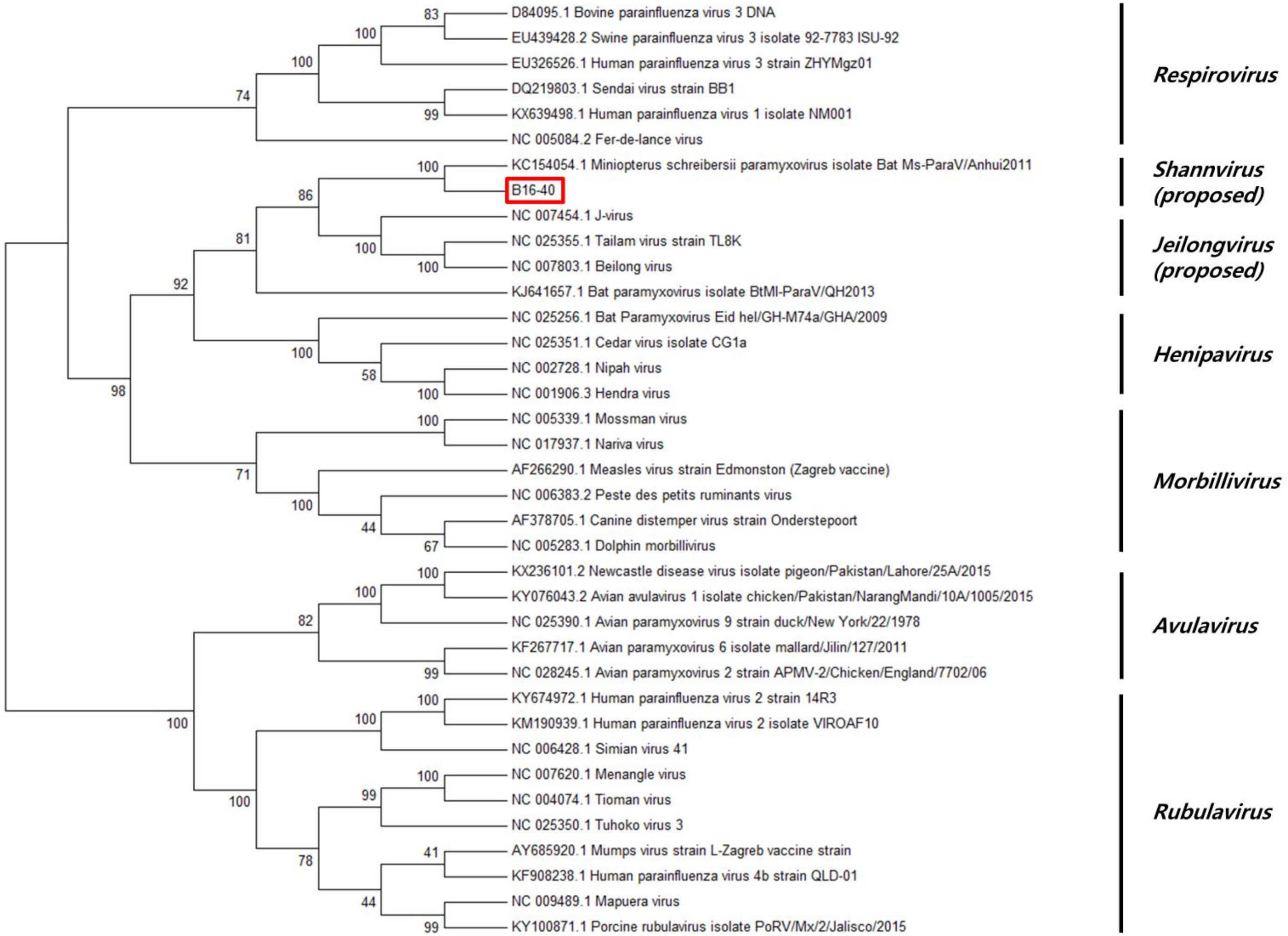

B. Fusion

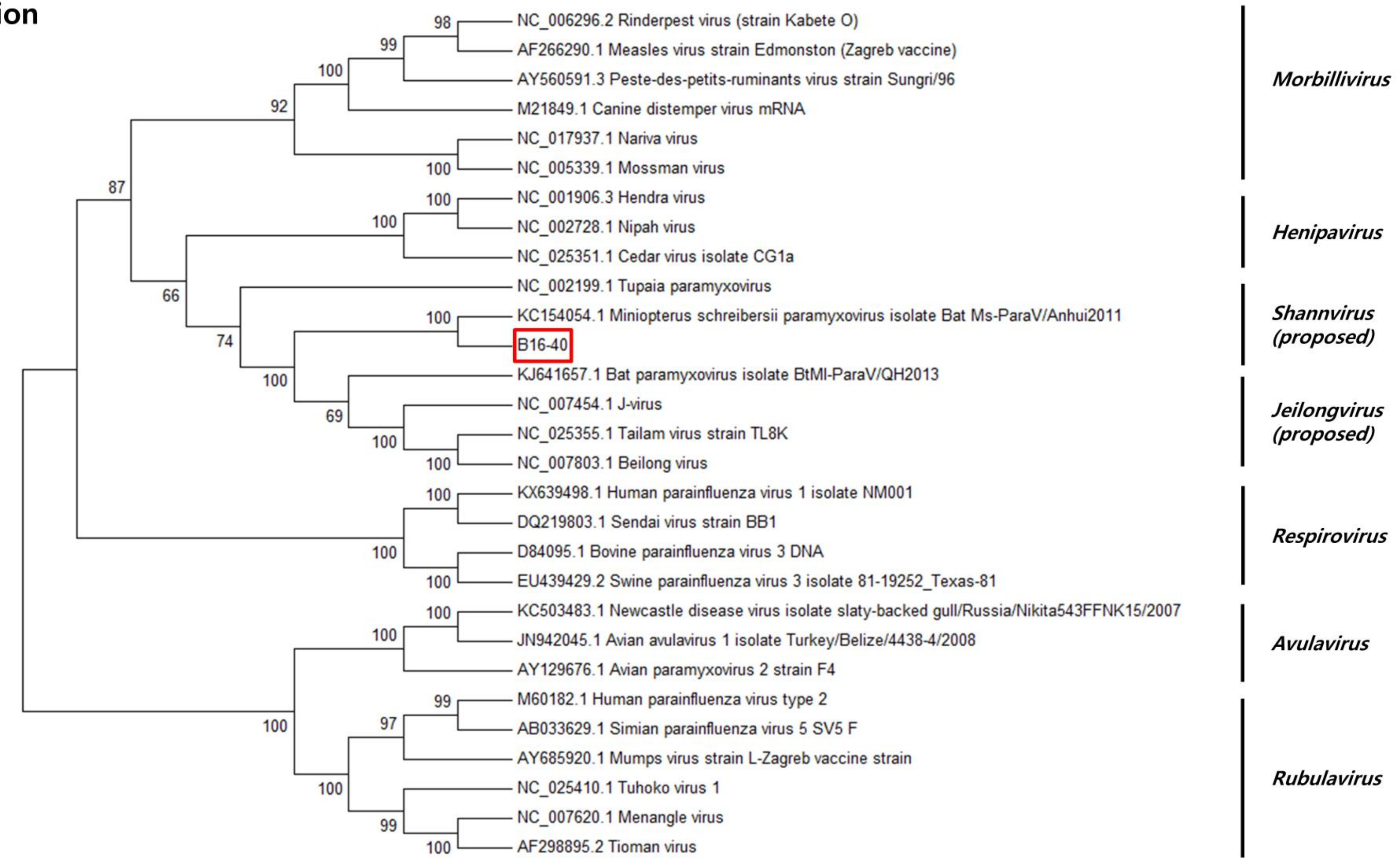
NC_006296.2 Rinderpest virus (strain Kabete O)
AF266290.1 Measles virus strain Edmonston (Zagreb vaccine)
AY560591.3 Peste-des-petits-ruminants virus strain Sungri/96
M21849.1 Canine distemper virus mRNA
NC_017937.1 Nariva virus
NC_005339.1 Mossman virus
NC_001906.3 Hendra virus
NC_002728.1 Nipah virus
NC_025351.1 Cedar virus isolate CG1a
NC_002199.1 Tupaia paramyxovirus
KC154054.1 Miniopterus schreibersii paramyxovirus isolate Bat Ms-ParaV/Anhui2011
B16-40
KJ641657.1 Bat paramyxovirus isolate BtMI-ParaV/QH2013
NC_007454.1 J-virus
NC_025355.1 Tailam virus strain TL8K
NC_007803.1 Beilong virus
KX639498.1 Human parainfluenza virus 1 isolate NM001
DQ219803.1 Sendai virus strain BB1
D84095.1 Bovine parainfluenza virus 3 DNA
EU439429.2 Swine parainfluenza virus 3 isolate 81-19252_Texas-81
KC503483.1 Newcastle disease virus isolate slaty-backed gull/Russia/Nikita543FFNK15/2007
JN942045.1 Avian avulavirus 1 isolate Turkey/Belize/4438-4/2008
AY129676.1 Avian paramyxovirus 2 strain F4
M60182.1 Human parainfluenza virus type 2
AB033629.1 Simian parainfluenza virus 5 SV5 F
AY685920.1 Mumps virus strain L-Zagreb vaccine strain
NC_025410.1 Tuhoko virus 1
NC_007620.1 Menangle virus
AF298895.2 Tioman virus
Morbillivirus
Henipavirus
Shannvirus (proposed)
Jeilongvirus (proposed)
Respirovirus
Avulavirus
Rubulavirus

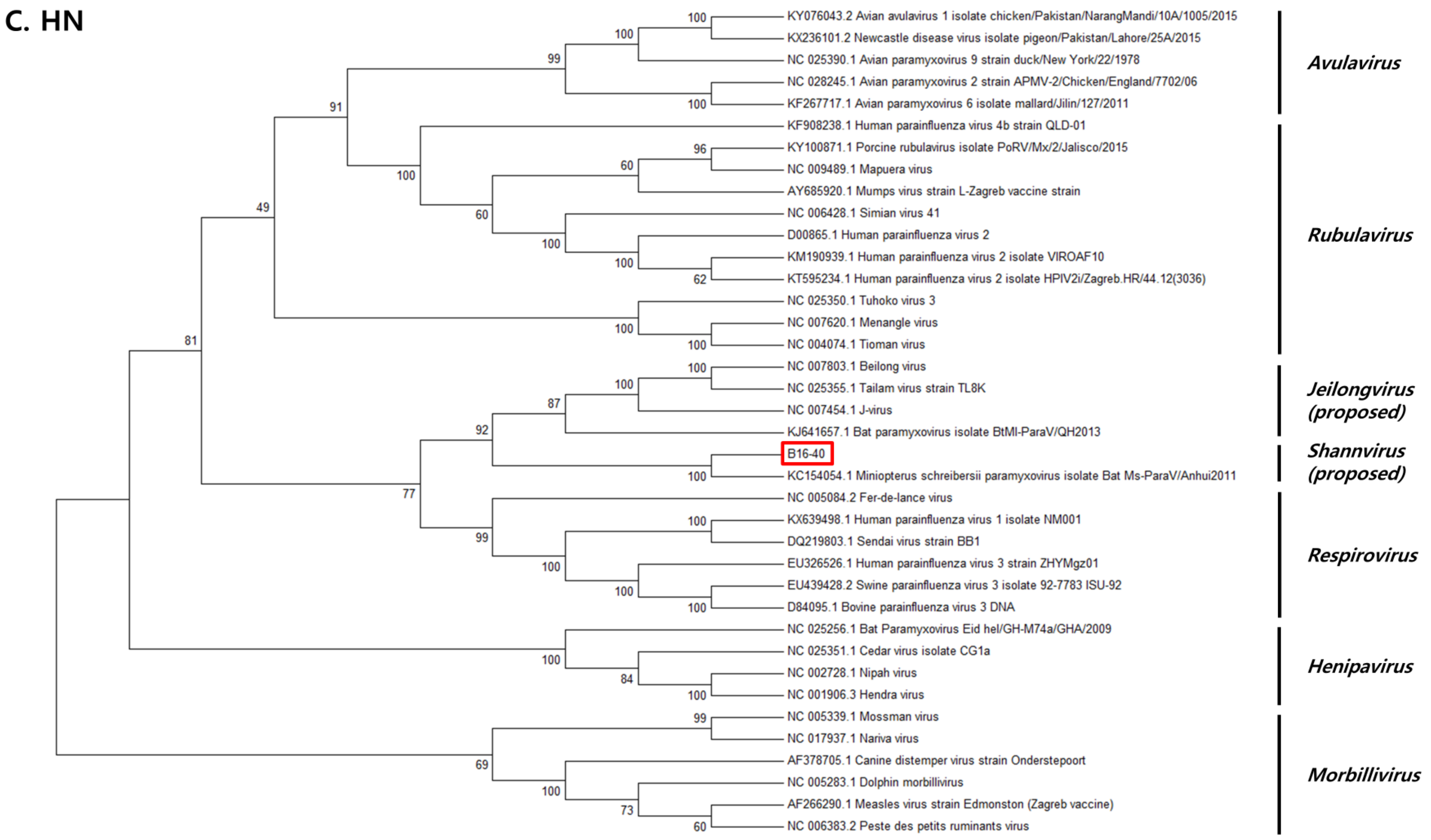
C. HN
KY076043.2 Avian avulavirus 1 isolate chicken/Pakistan/NarangMandi/10A/1005/2015
KX236101.2 Newcastle disease virus isolate pigeon/Pakistan/Lahore/25A/2015
NC 025390.1 Avian paramyxovirus 9 strain duck/New York/22/1978
NC 028245.1 Avian paramyxovirus 2 strain APMV-2/Chicken/England/7702/06
KF267717.1 Avian paramyxovirus 6 isolate mallard/Jilin/127/2011
KF908238.1 Human parainfluenza virus 4b strain QLD-01
KY100871.1 Porcine rubulavirus isolate PoRV/Mx/2/Jalisco/2015
NC 009489.1 Mapuera virus
AY685920.1 Mumps virus strain L-Zagreb vaccine strain
NC 006428.1 Simian virus 41
D00865.1 Human parainfluenza virus 2
KM190939.1 Human parainfluenza virus 2 isolate VIROAF10
KT595234.1 Human parainfluenza virus 2 isolate HPIV2i/Zagreb.HR/44.12(3036)
NC 025350.1 Tuhoko virus 3
NC 007620.1 Menangle virus
NC 004074.1 Tioman virus
NC 007803.1 Beilong virus
NC 025355.1 Tailam virus strain TL8K
NC 007454.1 J-virus
KJ641657.1 Bat paramyxovirus isolate BtMl-ParaV/QH2013
B16-40
KC154054.1 Miniopterus schreibersii paramyxovirus isolate Bat Ms-ParaV/Anhui2011
NC 005084.2 Fer-de-lance virus
KX639498.1 Human parainfluenza virus 1 isolate NM001
DQ219803.1 Sendai virus strain BB1
EU326526.1 Human parainfluenza virus 3 strain ZHYMgz01
EU439428.2 Swine parainfluenza virus 3 isolate 92-7783 ISU-92
D84095.1 Bovine parainfluenza virus 3 DNA
NC 025256.1 Bat Paramyxovirus Eid hel/GH-M74a/GHA/2009
NC 025351.1 Cedar virus isolate CG1a
NC 002728.1 Nipah virus
NC 001906.3 Hendra virus
NC 005339.1 Mossman virus
NC 017937.1 Nariva virus
AF378705.1 Canine distemper virus strain Onderstepoort
NC 005283.1 Dolphin morbillivirus
AF266290.1 Measles virus strain Edmonston (Zagreb vaccine)
NC 006383.2 Peste des petits ruminants virus
Avulavirus
Rubulavirus
Jeilongvirus (proposed)
Shannvirus (proposed)
Respirovirus
Henipavirus
Morbillivirus

Supplementary figure S2. **A.** Amino acid sequence alignment of the HN proteins from the human parainfluenza virus 1 (KBPV-VR-44) and bat paramyxovirus (B16-40) using the BioEdit program. The sialidase superfamily domain predicted by the NCBI Protein BLAST search is indicated by red arrows (https://blast.ncbi.nlm.nih.gov/Blast.cgi?PROGRAM=blastp&PAGE_TYPE=BlastSearch&LINK_LOC=blasthome). The conserved region of at least six amino acids in the HN amino acid sequences are indicated by red boxes. Neuraminidase activate sites (R, D, E, R, R, Y, and E) are indicated by blue arrows.[3-7] **B.** The location of the conserved amino acid sequences in the HN protein structure visualized and merged using the Chimera 1.11.2 program. The gold-colored structure is predicted from the HN protein sequence of the human parainfluenza virus 1 (KBPV-VR-44), and the blue-colored one is predicted from the bat paramyxovirus B16-40. The conserved regions of at least six amino acids are indicated with the corresponding conserved sequences in five different colors (red, yellow, green, blue, and purple).[8]

A. Amino acids sequence alignment of HN proteins between hPIV1 and bat paramyxovirus B16-40

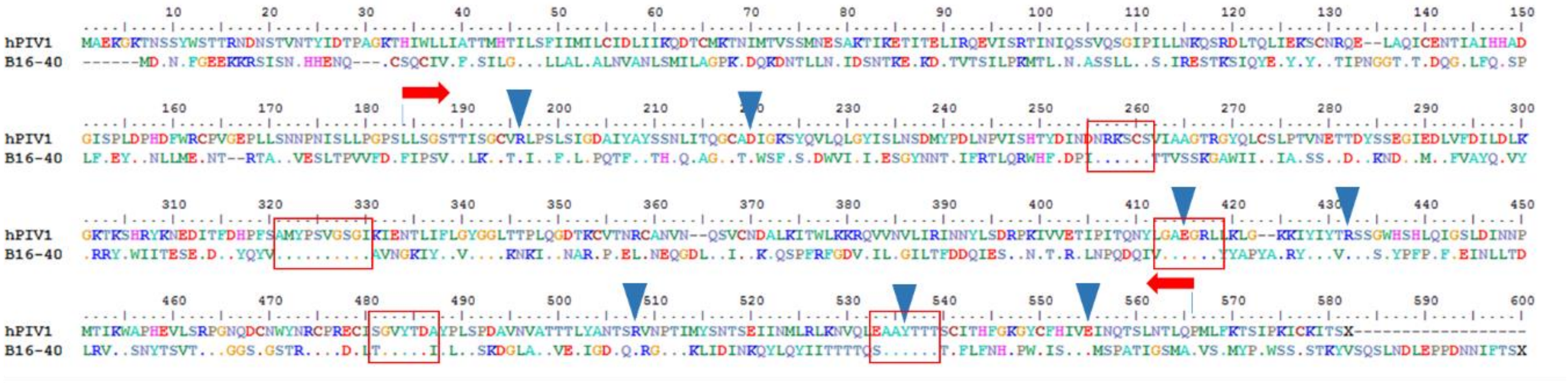

- ► Red arrows indicate sialidase superfamily domain predicted by NCBI Protein BLAST search.
  : e-value : 2.94e-146, cl21531
- ► Red Boxes indicates conserved sites (more than six amino acids)
- ► Blue arrows indicates neuraminidase activate sites (R D E R R Y E)

B. Location of the conserved Amino acids sequences on the HN proteins structure

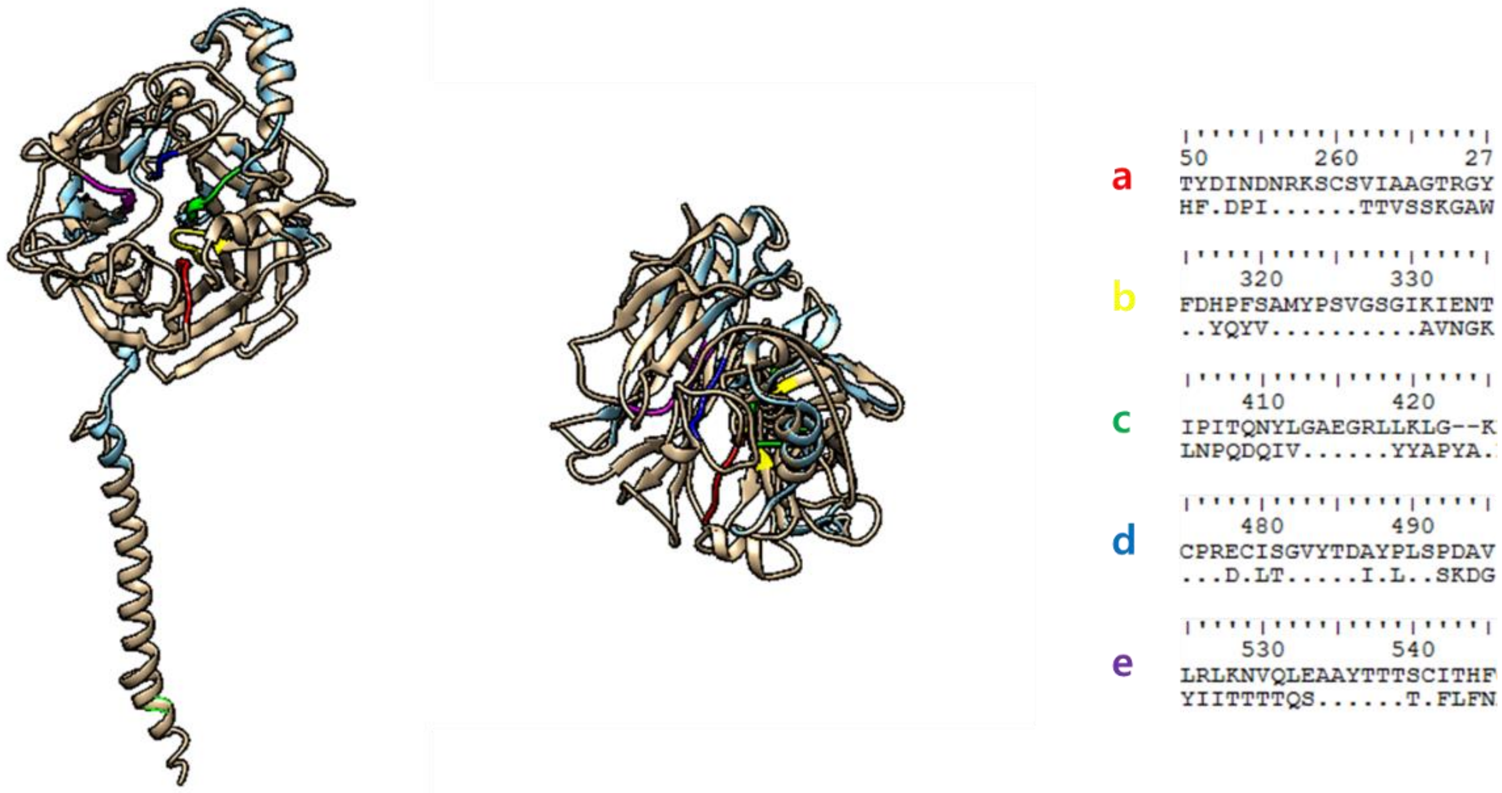

► HN structures of bat paramyxovirus B16-40 and human parainfluenza virus 1 were predicted using Phyre2 web portal, and visualized and merged using Chimera 1.11.2 program.

► Gold-colored structure predicted from human parainfluenza virus 1 (KBPV-VR-44) and Blue-colored one done from bat paramyxovirus B16-40

References

1. Katoh K, Standley DM. MAFFT multiple sequence alignment software version 7: Improvements in performance and usability. Mol Biol Evol. 2013;30.4:772–780. https://doi.org/10.1093/molbev/mst010

2. Tamura K, Stecher G, Peterson D, Filipski A, Kumar S. MEGA6: Molecular Evolutionary Genetics Analysis Version 6.0. Mol Biol Evol. 2013;30:2725–2729. https://doi.org/10.1093/molbev/mst197

3. Thompson, J. D., Higgins, D. G., & Gibson, T. J. CLUSTAL W: improving the sensitivity of progressive multiple sequence alignment through sequence weighting, position-specific gap penalties and weight matrix choice. Nucleic acids research. 1994;*22*(22):4673-4680. https://doi.org/10.1093/nar/22.22.4673

4. Marchler-Bauer, A., Bo, Y., Han, L., He, J., Lanczycki, C. J., Lu, S., et al. CDD/SPARCLE: functional classification of proteins via subfamily domain architectures. Nucleic acids research. 2016;45(D1): D200-D203. https://doi.org/10.1093/nar/gkw1129

5. Marchler-Bauer, A., Derbyshire, M. K., Gonzales, N. R., Lu, S., Chitsaz, F., Geer, L. Y., et al. CDD: NCBI's conserved domain database. Nucleic acids research. 2014;43(D1):D222-D226. https://doi.org/10.1093/nar/gku1221

6. Marchler-Bauer, A., Lu, S., Anderson, J. B., Chitsaz, F., Derbyshire, M. K., DeWeese-Scott, C., et al. CDD: a Conserved Domain Database for the functional annotation of proteins. Nucleic acids research. 2010;39(suppl_1):D225-D229. https://doi.org/10.1093/nar/gkq1189

7. Marchler-Bauer, A., & Bryant, S. H. CD-Search: protein domain annotations on the fly. Nucleic acids research. 2004;32(suppl_2):W327-W331. https://doi.org/10.1093/nar/gkh454

8. Kelley, L. A., Mezulis, S., Yates, C. M., Wass, M. N., & Sternberg, M. J. The Phyre2 web portal for protein modeling, prediction and analysis. Nature protocols. 2015;10(6): 845-858. https://doi.org/10.1038/nprot.2015.053
